# The effect of eucalyptus inhalation on pain and the quality of life in rheumatoid arthritis

**DOI:** 10.1016/j.conctc.2022.100976

**Published:** 2022-08-21

**Authors:** Zahra Kord Varkaneh, Arezou Karampourian, Khodayar Oshvandi, Zahra Basiri, Younes Mohammadi

**Affiliations:** a Department of Medical Surgical Nursing, School of Nursing and Midwifery, Hamadan University of Medical Sciences, Hamadan, Iran; b Urology and Nephrology Research Center, Chronic Diseases (Home Care) Research Center, Department of Medical Surgical Nursing, School of Nursing and Midwifery, Hamadan University of Medical Sciences, Hamadan, Iran; c Chronic Diseases (Home Care) Research Center, Department of Medical Surgical Nursing, School of Nursing and Midwifery, Hamadan University of Medical Sciences, Hamadan, Iran; d Department of Internal Medicine, School of Medicine, Shahid Beheshti Medical Educational Center, Hamadan University of Medical Sciences, Hamadan, Iran; e Department of Epidemiology, School of Public Health, Modeling of Non Communicable Diseases Research Center, Health Sciences & Technology Research Institute, Hamadan University of Medical Sciences, Hamadan, Iran

**Keywords:** Aromatherapy, Chronic pain, Quality of life, Complementary therapies, Arthritis rheumatoid

## Abstract

**Problem considered:**

Pain is one of the most significant symptoms of rheumatoid arthritis that reduce the quality of life. The purpose of the study was to determine the effect of eucalyptus on pain and the quality of life in patients with rheumatoid arthritis.

**Methods:**

In this randomized clinical trial, 70 patients with rheumatoid arthritis were selected by random sampling. In the eucalyptus group, 1 mL of eucalyptus oil was inhaled for 5 min, 3 times a day, for one month. The control group received placebo inhalation. Both groups used routine treatments. Data were collected using a questionnaire of demographics, the numerical pain rating scale (NRS), and Quality of Life (SF-12). Statistical analysis was done using 19th edition SPSS software and applied on paired *t*-test, chi-square, Fisher's exact test, and analysis of covariance.

**Results:**

The mean score of pain severity in the eucalyptus group significantly decreased in comparison with the control group (P < 0.001). The severity of pain there was no statistical difference in both groups before, the first, and the second weeks after the intervention, (p > 0.05); however, in the third and fourth weeks after the intervention, the mean severity of pain in the eucalyptus group was lower than in the control group, and these differences were statistically significant between the two groups (p < 0.05). Also, the patients' quality of life in the eucalyptus group was increased significantly (P < 0.001).

**Conclusion:**

The eucalyptus leads to pain reduction, and consequently, improves the quality of life of patients with rheumatoid arthritis.

**Trial registration:**

IRCT20160110025929N15 Registration date: **2018-10-07**; https://en.irct.ir/trial/33573.

## Abbreviations

**RA**: Rheumatoid Arthritis
**DMARDs**: Disease-Modifying Anti Rheumatic Drugs

## 1 Introduction

Rheumatoid arthritis is a chronic and inflammatory joint disease [1,2], the most common musculoskeletal disorder [2], and the most debilitating autoimmune disease with unknown origin [3,4]. In the 2010 study, the universal prevalence of RA was % 0.24. The disease occurs in women more than men [5]. Factors involved in this disease are smoking, gender, bacteria, and viruses [6]. This disease has articular and non-articular symptoms [7,8]. Pain is the main symptom of rheumatoid arthritis [5]. Pain reduces people's life quality [8], therefore pain control is one of the most important healthcare services in patients [9]. The quality of life is a multidimensional concept that has an impact on the physical, mental, social, and personal beliefs of individuals. Measuring the quality of life helps nurses to take care of people, whose life quality degraded significantly [10]. The study of Kim showed the significant impact of aromatherapy on pain and depression reduction but didn't lead to a higher quality of life [9]. Treatment of patients with RA requires a multidisciplinary approach [11]. Various therapeutic interventions such as pharmaceutical, non-pharmaceutical, and surgical interventions have been used for pain reduction in rheumatoid arthritis [12]. Pharmaceutical interventions may have undesirable complications such as severe cardiovascular and gastrointestinal problems, nephrotoxicity, and systemic infections [13]. The American College of Rheumatology has recommended guidelines for using disease-modifying anti-rheumatic drugs (DMARDs) to improve symptoms and restore joint function [14]. These drugs have countless predictable side effects [14]. Surgery is not always necessary and can lead to serious complications such as infection and bleeding [15]. Complementary medicine can also be used with DMARDs [5]. The patients have turned their attention to complementary medicine because of the side effects of pharmaceutical interventions [16]. Aromatherapy is one of the methods of complementary medicine [17]. Today, aromatherapy is accepted as holistic nursing care and part of professional nursing [10]. Eucalyptus is one of the herbal medicines used for rheumatoid arthritis patients. Different species of the plant are distributed all over the world [17] Eucalyptus has anti-inflammatory, anti-nociceptive and, anti-microbial properties. Eucalyptus, as analgesic, can be used topically or inhaled. It stimulates neurotransmitters and endorphin secretion inside the brain [18,19]. Eucalyptus inhalation is effective for relieving knee pain [20]. Due to limited studies about an analgesic and anti-inflammatory impacts of eucalyptus, researchers decided to perform a study about this topic. The goal of the current study is to identify the impact of eucalyptus inhalation on the pain and the quality of life of patients with RA.

## 2 Method and methods

**Fig. 1:**
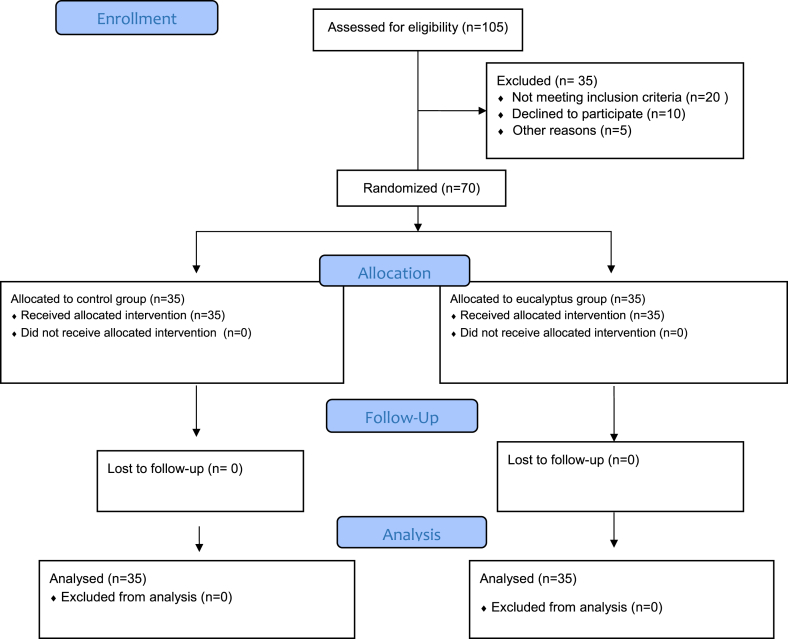
CONSORT 2010 flow diagram.

The study was a randomized clinical trial. The project was conducted on patients with RA, referred to the clinic (Fig. 1). In this randomized clinical trial, 70 patients with rheumatoid arthritis were selected by random sampling and were randomly divided into two groups. Subjects were selected by simple sampling method using R software version 3.5.1 and in the form of permutable blocks ABC-ACB -BCA -BAC -CAB -CAB is randomly assigned to two (eucalyptus and control). A sequence of blocks was randomly generated using R software and a list was created. Patients were randomly assigned to one of three groups based on the list. The patients did not know their assigned group, the eucalyptus' aroma could impact on the patients in control group. So, the patients were excluded if they were in the same room.

Based on the following formula, the number of patients in each group was estimated at 29 patients, however, considering of 15% of the sample attrition, 35 patients were invited in each group.

$$\frac{\left({\left(1.96+0.84\right)}^{2}\times 2\times 2^{2}\right)}{.1.5^{2}}=29$$

The inclusion criteria were age 18–60 years, no use of aromatherapy in the past month, absence of a history of allergy, allergic rhinitis, asthma, and chronic pulmonary disease, having at least one year of the definitive diagnosis of articular rheumatism. Exclusion criteria included allergy to eucalyptus, changing the disease process, death, and the patient migrates to another place. The primary and secondary outcomes were pain and quality of life.

The research tools were:

1 Demographic and clinical questionnaires include age, gender, job, education, marital status [21], duration of decease, location, smoking, time of diagnosis of rheumatoid arthritis, having Family history of rheumatoid arthritis, criterion DSA28, movement limitation, and BMI.
2 The numerical pain rating scale (NRS), the numerical pain scale with the values of 0 (no pain) to 10 (severe pain) was also used to record the pain severity. The patients recorded the pain before and after the intervention [15,22].
3 The Quality of Life (SF-12), the SF-12 questionnaire consisting of 8 subscales, the general perception of personal health, physical performance, physical health, emotional disorders, physical pain, social performance, joy, having energy for life, and mental health was used to measure. The reliability of this scale has been previously calculated based on the “test-retest”. Cronbach's alphas were respectively 0.86 and 0.76, for physical and mental dimensions, respectively [15,23]. In this study, the internal consistency of physical and mental dimensions of QOL was achieved equal 0.79 and 0.75 correspondingly. They were finally answered based on the Likert scale. For each sub-scale, a score of 0–100 was obtained. A higher score is indicative of better performance or well-being [24]. Their life quality was measured by the SF-12 questionnaire at first and at the end of the study.

In this study, the validity of the tools was confirmed by 10 nursing faculty members.

Interventions:

After obtaining informed consent for data collection, patients were randomly divided into control and eucalyptus groups.

1 Eucalyptus oil was prepared by a reputable pharmaceutical company in 2019. The essential oil used in the present study is from the species of Myrtassa which was prepared from the leaves of the plant. In the eucalyptus group, eucalyptus essential oil was placed on a 2 * 4- inch gas pad that attached to the patient's clothing with pins and was incubated for 5 min, 3 times a day. Pain score was measured 2 min later.
2 In the control group was used distilled water instead of eucalyptus. Both groups received medication as prednisolone (once a day), indomethacin (twice a day), celecoxib (twice a day), methotrexate (three numbers a week), and acetaminophen (once a day). Also, the eucalyptus group used eucalyptus as complementary medicine.

Data were analyzed using 19th edition SPSS software and paired *t*-test, chi-square, Fisher's exact test, and analysis of covariance. The study protocol was approved by the Ethics Committee with the number of IR-UMSHA.REC.1397.408. The IRCT code was IRCT20160110025929N15.

## 3 Results

The findings of this research showed that most of patients were women, married, illiterate, urban, non-smoking, having more than 4 years RA, having 3–4 joints involved based on criterion DSA28, and movement limitation. The two groups were similar in terms of the time of diagnosis, family history of rheumatoid arthritis, and movement limitation (Table 1).

**Table 1:** Demographic data in Eucalyptus and control groups.

Variables		Control group n = 35	Eucalyptus group n = 35	p-value
		N (%)	N (%)	
Gender, n (%)	Female	29(82.86)	29(82.86)	X^2^ = 1.47, p = 0.861^a^
	Male	6(17.14)	6(17.1)	
Age, M± SD		47.40 ± 18.57	52.91 ± 16.73	X^2^ = 1.31, p = 0.196^a^
Marital status, n (%)	Unmarried	6(17.14)	3(8.57)	X^2^ = 2.91, p = 0.364^a^
	Married	27(77.14)	29(82.86)	
	Other	2(5.72)	3(8.57)	
Education, n (%)	Illiterate	15(42.86)	16(45.71)	X^2^ = 0.82, p = 0.923^b^
	High school	12(34.28)	12(34.29)	
	Diploma	7(20.00)	6(17.14)	
	Academic	1(2.86)	1(2.86)	
Location, n (%)	Urban	24(68.57)	27(77.14)	X2 = 0.65, p = 0.420^b^
	Village	11(31.43)	8(22.86)	
Having Smoking, n (%)	Cigarette	0(0.00)	2(5.72)	X^2^ = 1.93, p = 0.743^a^
	Narcotics	1(2.86)	1(2.86)	
	None	34(97.14)	32(91.42)	
Time of diagnosis of rheumatoid arthritis, n (%)	≤4 year	17(48.57)	16(45.71)	X^2^ = 3.86, p = 0.445^a^
	>4 years	18(51.43)	19(54.29)	
Having Family history of rheumatoid arthritis, n (%) –		9(25.71)	3(8.57)	X^2^ = 3.62, p = 0.052^b^
Criterion DSA28, n (%)	1–2	9(25.71)	7(20.00)	X^2^ = 2.91, p = 0.321^b^
	3–4	13(37.14)	17(48.57)	
	5–6	5(14.29)	1(2.86)	
	>6	8(22.86)	10(28.57)	
Movement limitation, n (%)		22(62.86)	19(54.29)	X^2^ = 0.53, p = 0.467^b^
BMI, M± SD		22.63 ± 4.56	25.11 ± 5.18	X^2^ = 2.13, p = 0.037^a^

a Chi-square.

b Fisher's Exact Test.

The result showed that there was no statistical difference between the mean scores of the severity of pain in both groups before the study, (P = 0.580); also the first (P = 0.530), and the second weeks after the intervention (P = 0.458); however, at the third weeks (P = 0.003) and the fourth weeks after the intervention (P = 0.001) there was a statistical difference in both groups (Table 2).

**Table 2:** Comparison of severity of pain before and after the intervention in Eucalyptus and control groups.

Groups	Control n=35	Eucalyptus n=33	Tests	p-value
Intervention time				
Before the intervention, M ± SD	6.26 ± 1.63	6.46 ± 1.72	X^2^ = 0.56	0.580^a^
First week after the intervention, M ± SD	6.14 ± 1.65	6.40 ± 1.72	X^2^ = 0.64	0.530^a^
Second week after the intervention, M ± SD	5.68 ± 1.53	6.31 ± 4.75	X^2^ = 0.75	0.458^a^
Third week after the intervention, M ± SD	5.83 ± 1.56	4.65 ± 1.61	X^2^ = 3.09	0.003^a^
Fourth week after the intervention, M ± SD	6.00 ± 1.50	4.40 ± 1.58	X^2^ = 4.35	0.001^a^
Test statistic	F = 3.72 P = 0.016	F = 7.33 P = 0.010		

a Chi-square.

Also, the results showed that there was no statistical difference between the quality of life in both groups before the study (P = 0.5); however, after the intervention, there was a statistical difference between the quality of life in both groups (p < 0.001), so that total quality of life to increase from 28.26 ± 3.23 to 37.51 ± 4.57 in eucalyptus group (Table 3).

**Table 3:** Comparison of the quality of life before and after intervention in Eucalyptus and control groups.

Groups Dimensions Of Quality Of Life		Control n = 35	Eucalyptus n = 33	p-value
Total Physical Health, M ± Sd	before	10.91 ± 1.35	11.48 ± 2.07	0.603^a^
	after	9.66 ± 2.25	16.46 ± 1.87	0.001^a^
Total Mental Health, M ± Sd	before	17.68 ± 2.71	16.77 ± 1.80	0.345^a^
	after	14.48 ± 2.85	21.06 ± 2.90	0.001^a^
Total Quality Of Life, M ± Sd	before	28.60 ± 3.32	28.26 ± 3.23	0.431^a^
	after	24.14 ± 4.51	37.51 ± 4.57	0.001^a^
Test statistic		p = 0.016	p = 0.010	

a Chi-square.

## 4 Discussion

According to the results of the current study, it can be argued that the pain of patients who used eucalyptus oil inhalation was significantly reduced. The study of Kim showed that the anxiety reduced significantly after eucalyptus inhalation (p < 0.001) in comparison to the control group [9]. Massage therapy has been shown to reduce fatigue as well as the intensity and quality of pain in patients. Since the control group required pain-relieving methods, so they used pain-relieving methods such as massage, joint surgery, joint replacement and, joint injection more than others. Both groups were identical in terms of drug and surgical treatments before intervention. The control group after the intervention used other relief methods such as massage, oil massage, and warm compress. The results of the current study showed that the mean scores of all dimensions of life quality in the intervention group were more than those of the control group (P < 0.001). Aromatherapy by anti-inflammatory oils leads to reduction of pain of patients, joyful feeling, and improvement of daily activities. The quality of life and life expectancy also increases. No previous study examined the impact of eucalyptus oil on the life quality of rheumatoid arthritis patients. The study of Kuçukdeveci showed that medical herbs with anti-inflammatory impact can reduce pain and improve patients' quality of life [25].

Since one of the limitations of this study was the small number of samples due to time constraints, it is recommended that future studies be performed with more samples. Another limitation of this study was the low follow-up time to assess the quality of life. It is also suggested that studies be carried out with longer periods. Also despite instructing the patient not to use other complementary medicine methods, it was possible that some patients had used other non-pharmacological interventions.

## 5 Conclusion

The results of this study showed that eucalyptus oil reduced pain and increased quality of life. Therefore, along with other treatments, eucalyptus oil can also be used as a complementary treatment.

## Authors' contribution

Kord-Varkaneh, Karampourian, Oshvandi made substantial contributions to the conception and design of the study. Sampling was carried out under the supervision of Basiri. Data analysis was performed by Mohammadi. Kord-Varkaneh and Karampourian were involved in the writing-up of the manuscript. All read and approved the final manuscript.

## Ethics approval and consent to participate

The study protocol was approved by the Ethics Committee with the number 9708224883 - IR-UMSHA.REC.1397.408. The IRCT code was IRCT20160110025929N15.

## Consent for publication

Authors are given permission to publish the manuscript.

## Fund

Hamadan University of Medical Sciences, Hamadan, Iran.

## Declaration of competing interest

The authors have no conflicts of interest to declare.

## Data Availability

Data will be made available on request.
